# Impact of genetically predicted atrial fibrillation on cancer risks: A large cardio-oncology Mendelian randomization study using UK biobank

**DOI:** 10.3389/fcvm.2022.974402

**Published:** 2023-01-05

**Authors:** Wenjie Li, Mingkai Huang, Rong Wang, Wei Wang

**Affiliations:** ^1^Department of Radiation Oncology, Nanfang Hospital, Southern Medical University, Guangzhou, China; ^2^Department of Cardiology, Union Hospital, Tongji Medical College, Huazhong University of Science and Technology, Wuhan, China

**Keywords:** cancer, Mendelian randomization, single-nucleotide polymorphism, atrial fibrillation, prevention

## Abstract

**Background:**

Increasing incidences of both atrial fibrillation (AF) and cancer have been observed in recent years. However, the casual association of both serious conditions has been scarcely evaluated and is considered to be a blank slate in cardio-oncology. Thus, we introduced Mendelian randomization (MR) methods to estimate the effects of AF on cancer risks.

**Methods:**

We performed univariable and multivariable two-sample MR analyses to evaluate the effects of AF on the risk of 19 site-specific types of cancer. This MR study was conducted based on 111 independent AF-associated genetic instruments from genome-wide association studies and summarized-level data from corresponding cancer consortia. Multiple sensitivity analyses, including the leave-one-out analysis, MR-Egger regression, and MR-PRESSO tests, were further performed to examine the potential directional pleiotropic effects. Functional annotation was performed for common differentially expressed genes of AF and prostate cancer (PCA).

**Results:**

A total of 6,777,155 European-descent people, including 533,725 cases and 6,243,430 controls, were included in the present MR analysis. Univariable MR analyses demonstrated a causal effect of AF on the incidence of PCA [odds ratio (OR): 0.96; 95% confidence interval (CI) 0.92–0.99, *p* = 0.01], and the causal effect remained significant (OR: 0.65; 95% CI 0.47–0.90, *p* = 0.01) after adjusting for potential confounders through the multivariable MR approach. However, no casual associations between AF and the other 18 site-specific cancer risks were observed (all *p*-values were > 0.05). The consistency of outcomes across complementary sensitivity MR methods further supported the causality. The functional analysis emphasized the essential role of antioxidant and xenobiotic catabolic processes in AF and PCA.

**Conclusion:**

Contrary to the findings of several previous observational studies, our comprehensive MR analyses did not corroborate a causal role for AF in increasing the risk of various types of cancer. They did, however, demonstrate that AF may decrease the risk of PCA. Studies from larger sample sizes and individuals with different ethnic backgrounds are required to further support our conclusions.

## 1. Introduction

Atrial fibrillation (AF) is the most common cardiac arrhythmia (1) that imposes a substantial risk of death from many cardiovascular diseases and huge societal healthcare burdens. It is presumed that 6–12 million people will develop AF in the USA by 2050 and 17.9 million citizens in Europe by 2060 (2). Interestingly, a recent prospective cohort study has reported that cancer is the leading causes of death in high-income countries, accounting for two times as many deaths as cardiovascular diseases (3). Moreover, abundant evidence based on large populations shows that patients with cancer are related to an increased incidence of AF (4). However, the effect of AF on cancer risks is an uncharted field in cardio-oncology (5). Given the unacceptably high prevalence and treatment costs of both serious conditions, cardio-oncology should not be only concerned with the cardiac side effects of antineoplastic drugs in this case (6). Thus, a specific study is urgently required to find the potential associations between AF and cancer risks, which may provide new insights into the possible mechanisms and therapeutic targets.

Several observational studies have described the ambiguous effects of AF on types of cancer (7–12) due to shared risk factors and predisposing biological processes (13, 14). Prior evidence shows that patients with new-onset AF would have a noticeably increased risk of a malignant diagnosis (7), which was consistent with those of population-based cohort studies that showed that AF was related to a higher malignant incidence (8, 9, 11). However, several intrinsic methodological limitations in prior study designs may impact the observed results, thus resulting in contradictory conclusions. It is difficult, for example, for observational studies to rule out several important lifestyle differences or residual confounders (e.g., smoking, alcohol consumption, diabetes, or hypertension) in both AF and types of cancer (14). Moreover, observational results suggest that an observed association might be attributable to instances associated with a cancer diagnosis and detection bias instead of a causal relationship (9). Consequently, there is an urgent need for a reliable study design that will assess the exact causal association between AF and cancer risks.

Mendelian randomization (MR) analysis has recently become a promising and novel epidemiological approach to assess the causal relationship between exposures and outcomes, using genetic variants as instrumental variables (IVs). Adopting genetic variants as the IVs in the MR analysis can make it less susceptible to reverse causality and hypothetical confounders. A two-sample MR analysis can be performed with robust statistical power using summary-level data from large genome-wide association studies (GWAS) (15). Hence, to solve the aforementioned issue regarding AF and types of cancer, we aimed to evaluate the causal relationship between AF and the risks of 19 site-specific types of cancer with univariable and multivariable two-sample MR methods. Given the tight association between AF and heart failure (HF) (16), we also evaluated the causality between HF and cancer risks.

## 2. Method

### 2.1. Mendelian randomization assumptions

**Figure 1:**
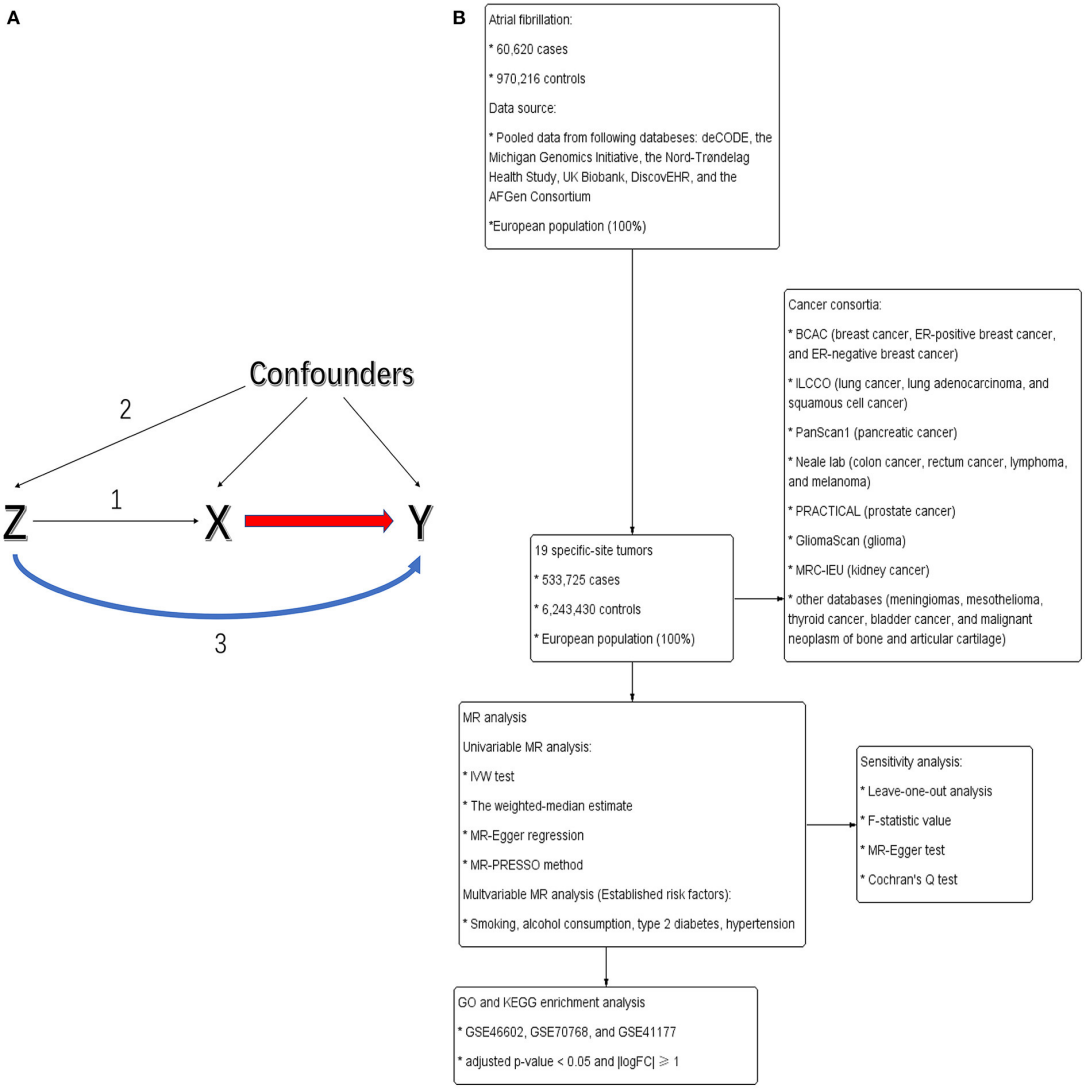
**(A)** Directed acyclic graph portraying the design of Mendelian randomization and potential pleiotropy. Genetic variants (Z) served as genetic instruments to evaluate whether exposures (X) were causally related to outcomes (Y). Numbers 1–3 represent assumptions 1–3. **(B)** The flowchart of this study.

To enable a valuable interpretation, all analyses in our MR study were based on the following three core hypotheses or study designs (17): (i) the IVs were convincingly correlated with exposures, (ii) the IVs influenced tumors only through their effects on exposures, and (iii) the IVs were independent of any confounders from the AF/HF cancer association (Figure 1A).

### 2.2. Selection of genetic instruments for exposures

The flowchart of this study is shown in Figure 1B. Exposures considered in this analysis included AF and HF. Variant AF/HF relationships were derived and manually extracted from the available summary-level GWAS. Nielsen et al. (18) carried out a GWAS with 60,620 AF cases and 970,216 controls to distinguish genetic variations. This study was mainly derived from six databases [deCODE, the Michigan Genomics Initiative (MGI), the Nord-Trøndelag Health Study (HUNT), UK Biobank, DiscovEHR, and the AFGen Consortium]. All enrolled patients were of European descent and generally elder people (the median age at first AF diagnosis: MGI: 65; deCODE: 74; HUNT: 76). The genetic variation of HF was derived from a GWAS meta-analysis of HF comprising 47,309 European-descent cases and 930,014 controls from the Heart Failure Molecular Epidemiology for Therapeutic Targets Consortium (19).

Given that the violation of three MR assumptions may lead to unreliable conclusions, the following steps would help choose the best IVs. First, we extracted accessible summary-level data from Nielsen et al. (18) and Sonia et al. (19) and set up a significance threshold of *p* < 5 × 10^−8^. Detailed information about AF/HF-related single-nucleotide polymorphisms (SNPs) is shown in Supplementary Table 1. To measure genetic correlation, we further conducted a linkage disequilibrium (LD) clumping test at an *R*^2^ < 0.001 and 10,000 kb window to preserve the SNPs that were most robustly associated with the AF/HF for downstream analysis. To exclude bias from weak instruments, the *F*-statistic value was assessed on the bias of the formula $F=\frac{R2\left(N-k-1\right)}{k\left(1-R2\right)}$, where *R*^2^ is the proportion of variance explained by the IVs, *k* and *N* are the number of SNPs and enrolled patients, respectively. *F*-statistic values > 10 were robust enough to avoid weak instrument bias. Eventually, several SNPs were excluded to eradicate the genetic bias created by palindromes with intermediate allele frequencies (20), and we created a total of 111 and 12 SNPs as the original AF- and HF-IVs, respectively.

### 2.3. Study participants of various types of cancer

To obtain genetic data for 19 site-specific tumors, we analyzed the following European cancer consortia: Breast Cancer Association Consortium (BCAC) (21) (breast cancer: 122,977 patients and 105,974 controls; ER-positive breast cancer: 69,501 patients and 105,974 controls; and ER-negative breast cancer: 21,468 patients and 105,974 controls), Prostate Cancer Association Group to Investigate Cancer Associated Alterations in the Genome (PRACTICAL) (22) [prostate cancer (PCA): 79,148 patients and 61,106 controls], International Lung Cancer Consortium (ILCCO) (23) (lung cancer: 11,348 patients and 15,861 controls, lung adenocarcinoma: 3,442 patients and 14,895 controls; and squamous cell cancer: 3,275 patients and 15,038 controls), PanScan1 (24) (pancreatic cancer: 1,896 patients, 1,939 controls), GliomaScan (25) (glioma: 6,811 patients, 1,856 controls), the Medical Research Council-Integrative Epidemiology Unit (MRC-IEU) consortium (kidney cancer: 1,114 patients, 461,896 controls; pancreatic cancer: 1,114 patients, 461,896 controls; rectum cancer: 1,470 patients, 461,540 controls), Neale lab (colon cancer: 2,437 patients, 358,757 controls; lymphoma: 1,752 patients, 359,442 controls; melanoma: 3,598 patients, 459,335 controls), and other databases (malignant neoplasm of bone and articular cartilage: 119 patients, 174,006 controls; thyroid cancer: 989 patients, 174,006 controls; bladder cancer: 1,279 patients, 372,016 controls; mesothelioma: 133 patients, 174,006 controls; meningiomas: 455 patients, 86,713 controls) (Table 1).

**Table 1:** Details of studies in the present Mendelian randomization analyses.

**Phenotype**	**Consortium**	**Sample size**	**Number of patients**	**Number of controls**	**Number of variants**	**GWAS Trait ID**	**Ethnicity**	**F-statistic**
**Exposure**								
Atrial fibrillation	NA	103,0836	60,620	970,216	33,519,037	ebi-a-GCST006414	European	NA
Heart failure	NA	977,323	47,309	930,014	77,73,021	ebi-a-GCST009541	European	NA
**Outcome**								
Overall cancer	UK Biobank	4,42239	70,223	3,72,016	12,321,875	ieu-b-4966	European	277.9
Breast cancer	BCAC	228,951	122,977	105,974	10,680,257	ieu-a-1126	European	281
ER+ breast cancer	BCAC	175,475	69,501	105,974	10,680,257	ieu-a-1127	European	281
ER– breast cancer	BCAC	127,442	21,468	105,974	10,680,257	ieu-a-1135	European	281
Lung cancer	ILCCO	85,716	29,266	56,450	89,45,893	ieu-a-966	European	281.4
Lung adenocarcinoma	ILCCO	66,756	11,273	55,483	88,81,354	ieu-a-965	European	281.4
Squamous cell lung cancer	ILCCO	63,053	7,426	55,627	88,93,750	ieu-a-967	European	281.4
Prostate cancer	PRACTICAL	140,254	79,148	61,106	19,733,911	ebi-a-GCST006085	European	273.8
Glioma	GliomaScan	6,811	1,856	4,955	309,636	ieu-a-1013	European	326
Kidney cancer	MRC-IEU	463,010	1,114	461,896	98,51,867	ukb-b-1316	European	275.6
Pancreatic cancer	MRC-IEU	46,3010	233	462,777	521,863	ieu-a-822	European	369.1
Rectum cancer	MRC-IEU	463010	1470	461540	9851867	ukb-b-1251	European	261.2
Lymphoma	Neale Lab	361,194	1,752	359,442	361,194	ukb-d-C_LYMPHOMA	European	273.8
Melanoma	Neale Lab	337,159	2,677	334,482	10,855,955	ukb-d-C3_MELANOMA_SKIN	European	273.8
Colon cancer	Neale Lab	36,1194	2,437	358,757	10,788,369	ukb-d-C3_COLON	European	349.6
Mesothelioma	NA	17,4139	133	174,006	16,380,303	finn-b-C3_MESOTHELIOMA_EXALLC	European	293.8
Meningiomas	NA	87,168	455	86,713	16,152,119	finn-a-CD2_BENIGN_MENINGES_EXALLC	European	NA
Thyroid cancer	NA	17,4995	989	174,006	16,380,316	finn-b-C3_THYROID_GLAND_EXALLC	European	293
Bladder cancer	NA	373,295	1,279	372,016	99,049,26	ieu-b-4874	European	290
Malignant neoplasm of bone and articular cartilage	NA	174,125	119	174,006	16,380,303	finn-b-C3_BONE_CARTILAGE_EXALLC	European	293.8

MR, Mendelian Randomization; GWAS, Genome Wide Association Study; BCAC, Breast Cancer Association Consortium; ILCCO, International Lung Cancer Consortium; PRACTICAL, Prostate Cancer Association Group to Investigate Cancer Associated Alterations in the Genome; MRC-IEU, Medical Research Council-Integrative Epidemiology Unit; NA, not available.

### 2.4. Statistical analysis of MR estimates

All MR analyses were performed in R 4.0.5 using the package TwoSampleMR (version 0.5.0).

#### 2.4.1. Two-sample MR method

We performed the inverse-variance weighted (IVW) test, which can provide a coherent estimation of the causality between genetically determined exposures and outcomes. It is made up of a meta-analysis of a single SNP's Wald ratio between the exposures and outcomes using a random-effects inverse-variance method, which can weigh every single Wald ratio according to its standard error to judge potential measurable heterogeneity (20). The causal effects were calculated and presented in the form of odds ratios (ORs) with 95% confidence intervals (CIs) for 14 site-specific types of cancer. Two-sided *p*-values < 0.05 were considered to be statistically significant. Of note, the results of IVW tests might be biased given the horizontal pleiotropy in invalid instrumental variables. Hence, the MR-Egger regression and the weighted-median estimate were conducted to predominantly assess the MR outcomes (20, 26). The MR-Egger regression can amend the IVW test by allowing a nonzero intercept that can provide an exploration of pleiotropy and an evaluation of the causality adjusted for pleiotropy (20). The weighted-median analysis is used to pool the median effects of all SNPs and can return an unbiased estimate once 50% of the SNPs are valid instruments (20). Finally, the MR Pleiotropy Residual Sum and Outlier (MR-PRESSO) test was conducted using the “MRPRESSO” R package to distinguish outlying SNPs that may result in horizontal pleiotropy and causal effects.

#### 2.4.2. Multivariable MR analysis

To support the univariate MR results and the third assumption, multivariable MR analyses adjusted for confounders, including smoking (trait ID: ukb-a-225), alcohol consumption (trait ID: ukb-d-20117_2), type 2 diabetes (trait ID: ebi-a-GCST006867), and hypertension (trait ID: ukb-b-14057) were introduced. Multivariable MR showed that the SNPs used in univariate MR analyses were also related to these confounders. Then, multivariable MR estimated the effects of each exposure on a single outcome. That is, this can simultaneously assess the effects of all risk factors that share a set of overlapping SNPs and make sure that the direct effects of each exposure on outcomes will not be mediated by other factors (27). As we included MR analyses of 19 site-specific types of cancer, a Bonferroni-adjusted *p-*value less than the threshold (i.e., 0.05/19 = 0.0026) was deemed as a significant causality to adjust for multiple-comparison tests. A potential relationship was considered significant if a *p*-value is between 0.05 and 0.0026.

### 2.5. Pleiotropy and sensitivity analysis

We conducted the leave-one-out analyses to assess whether the results of the IVW tests would be biased by single-sensitive SNPs (26). The aforementioned Egger intercept analysis was then performed to estimate the horizontal pleiotropy. The MR-heterogeneity analysis was ultimately performed to single out SNPs that were responsible for heterogeneity in casual estimation by means of Cochran's *Q*-test (28).

### 2.6. Identification and enrichment analyses of DEGs

Two PCA-related microarray datasets [GSE46602 (29) and GSE70768 (30)] and one AF-related dataset [GSE41177 (31)] were downloaded from the GEO (http://www.ncbi.nlm.nih.gov/geo) database to select differentially expressed genes (DEGs). Herein, genes with an adjusted *p*-value of < 0.05 and |logFC| ≥ 1 were considered DEGs. Gene Ontology (GO) and Kyoto Encyclopedia of Genes and Genomes (KEGG) pathway were performed to explore the potential biological functions of DEGs.

## 3. Results

**Figure 2:**
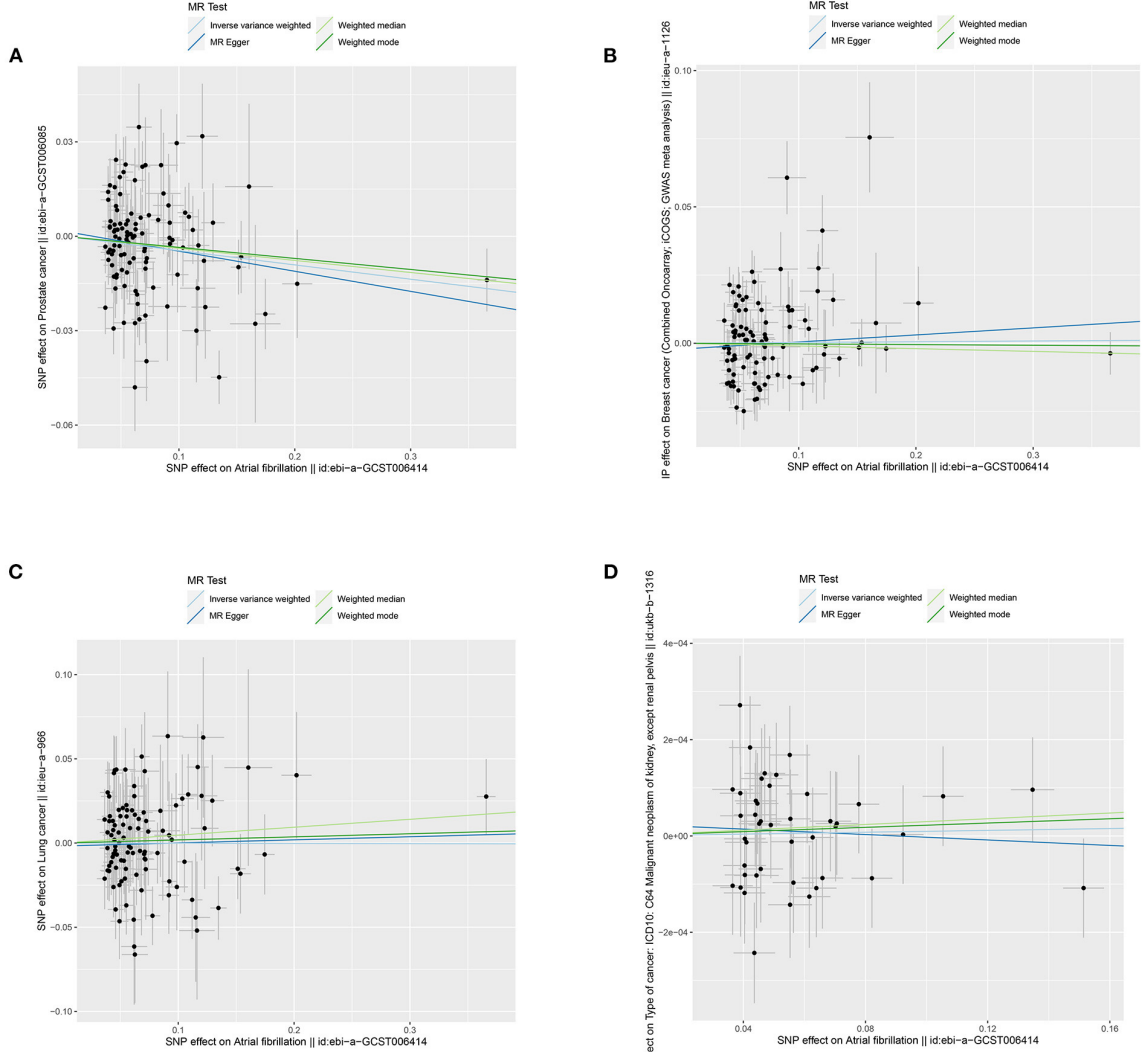
Scatterplots for MR analyses of the causal effect of atrial fibrillation on cancer risks: **(A)** prostate cancer; **(B)** breast cancer; **(C)** lung cancer; and **(D)** kidney cancer. The x-axis represents the previously published β-estimate for the association of each SNP with atrial fibrillation. The y-axis outlines the β-estimate for the relationship of each SNP with cancer risks by means of the multivariate logistic regression model. Lines represent causal estimates from the different methods. The slope of each line indicates the estimated MR effect per method. Circles correspond to marginal genetic associations with atrial fibrillation and risk of outcome for each variant. Error bars indicate 95% confidence intervals. SD, standard error; MR, Mendelian randomization.

In general, this MR study included a total of 6,334,916 European-descent people, with 463,502 cases and 5,871,414 controls (Table 1). Considering the variation in sample sizes of different cancers, *F*-statistics values in this study ranged from 273.8 to 369.1. The instruments (*F* > 100) used in our MR analyses were very strong to avoid bias (Table 1). All the MR evaluations for multi-polymorphism scores are shown in Tables 2, 3. Our results indicated that the genetically predicted AF was associated with a decreased risk of cancers of PCA and found no detrimental effects of AF/HF on the other 18 site-specific cancer risks (Tables 2, 3). The estimated effect sizes of the SNPs on both exposure (AF) and outcomes (PCA, breast cancer, lung cancer, and kidney cancer) are displayed in scatterplots (Figure 2).

**Table 2:** Mendelian randomization estimates of the casual relationships between atrial fibrillation and cancer risks.

**Exposure**	**nSNPs**	**IVW method**		**Weighted median method**		**MR–Egger**	
		**OR (95% CI)**	**P-value**	**OR (95% CI)**	**P-value**	**OR (95% CI)**	**P-value**
Overall cancer	107	1.0025 (0.9999–1.0052)	0.06	1.0017 (0.9974–1.0060)	0.4	1.0051 (0.9999–1.01)	0.06
Breast cancer	99	1.0026 (0.97–1.035)	0.87	1.00 (0.97–1.028)	0.87	1.026 (0.96–1.090)	0.41
ER+ breast cancer	99	0.99 (0.96–1.030)	0.76	0.98 (0.94–1.025)	0.42	1.014 (0.95–1.084)	0.69
ER– breast cancer	99	1.0089 (0.96–1.060)	0.73	0.99 (0.92–1.06)	0.7	1.07 (0.97–1.18)	0.16
Lung cancer	104	1.00 (0.94–1.060)	0.97	1.049 (0.96–1.14)	0.3	1.019 (0.91–1.14)	0.75
Lung adenocarcinoma	104	1.01 (0.93–1.10)	0.76	1.06 (0.92–1.20)	0.42	1.05 (0.90–1.24)	0.52
Squamous cell lung cancer	104	1.004 (0.92–1.10)	0.93	1.02 (0.88–1.18)	0.78	0.98 (0.83–1.17)	0.85
Prostate cancer	108	0.96 (0.92–0.99)	**0.01**	0.96 (0.92–1.0080)	0.11	0.94 (0.88–1.0040)	0.07
Glioma	50	1.15 (0.97–1.36)	0.12	1.22 (0.93–1.59)	0.14	1.24 (0.92–1.66)	0.16
Kidney cancer	44	1.000096 (0.9996–1.00060)	0.71	1.0003 (0.9996–1.001)	0.42	0.9997 (0.9984–1.0010)	0.67
Pancreatic cancer	52	0.87 (0.72–1.06)	0.16	0.83 (0.62–1.10)	0.19	0.78 (0.57–1.09)	0.15
Lymphoma	108	1.00016 (0.995–1.00049)	0.95	0.9995 (0.9987–1.00036)	0.27	0.9997 (0.9988–1.00063)	0.52
Melanoma	108	1.00036 (1.00–1.0010)	0.27	1.0010 (1.00–1.0020)	**0.045**	1.0011 (1.00–1.0020)	0.07
Colon cancer	108	0.9998 (0.9992–1.0004)	0.44	0.9990 (0.9980–1.00)	**0.041**	0.9995 (0.9983–1.0006)	0.39
Malignant neoplasm of bone and articular cartilage	106	0.78 (0.54–1.13)	0.2	0.71 (0.37–1.37)	0.31	0.75 (0.37–1.53)	0.43
Mesothelioma	106	1.24 (0.85–1.81)	0.27	1.25 (0.64–2.43)	0.51	1.02 (0.49–2.12)	0.96
Rectum cancer	61	1.002 (0.9997–1.00068)	0.5	1.009 (0.9994–1.00094)	0.61	1.000065 (0.9986–1.0015)	0.93
Meningiomas	83	0.95 (0.76–1.18)	0.65	0.90 (0.61–1.31)	0.57	0.87 (0.58–1.31)	0.50
Thyroid cancer	106	0.95 (0.84–1.09)	0.48	1.02 (0.8–1.3)	0.88	0.90 (0.70–1.15)	0.4
Bladder cancer	107	0.9999 (0.9996–1.00038)	0.9	0.9999 (0.9991–1.00065)	0.76	0.9993 (0.9986–1.00014)	0.11

The bold values are statistically significant (*p* < 0.05).

**Table 3:** Mendelian randomization estimates of the associations between heart failure and cancer risks.

**Exposure**	**nSNPs**	**IVW method**		**Weighted median method**		**MR–Egger**	
		**OR (95% CI)**	**P-value**	**OR (95% CI)**	**P-value**	**OR (95% CI)**	**P-value**
Overall cancer	9	0.99 (0.98–1.01)	0.56	1.0053 (0.99–1.02)	0.45	1.03 (0.99–1.07)	0.19
Breast cancer	9	0.93 (0.65–1.31)	0.66	1.065 (0.93–1.23)	0.38	1.35 (0.47–3.86)	0.59
ER+ breast cancer	9	0.95 (0.67–1.32)	0.75	1.00 (0.85–1.17)	0.96	1.31 (0.47–3.38)	0.62
ER– breast cancer	9	0.90 (0.61–1.34)	0.61	0.94 (0.69–1.28)	0.71	1.07 (0.31–3.66)	0.91
Lung cancer	9	1.0081 (0.75–1.35)	0.96	1.03 (0.75–1.41)	0.85	0.64 (0.28–1.44)	0.32
Lung adenocarcinoma	9	1.042 (0.64–1.70)	0.87	1.12 (0.67–1.86)	0.67	0.62 (0.15–0.59)	0.53
Squamous cell lung cancer	9	1.10 (0.77–1.56)	0.60	1.32 (0.83–2.11)	0.24	0.53 (0.20–1.44)	0.25
Prostate cancer	9	1.11 (0.92–1.35)	0.25	1.09 (0.94–1.28)	0.25	1.58 (0.94–2.66)	0.13
Glioma	6	0.78 (0.30–2.01)	0.61	1.33 (0.52–3.39)	0.55	3.88 (0.14–111.11)	0.47
Kidney cancer	2	0.9979 (0.9922–1.0036)	0.48	NA	NA	NA	NA
Pancreatic cancer	6	2.11 (0.41–10.87)	0.37	0.99 (0.37–2.64)	0.99	0.14 (0.00051–40.12)	0.54
Lymphoma	NA	NA	NA	NA	NA	NA	NA
Melanoma	9	1.00054 (0.9978–1.0034)	0.71	0.9996 (0.9959–1.0033)	0.82	1.0065 (0.9989–1.014)	0.14
Colon cancer	9	0.9984 (0.9959–1.00092)	0.21	0.9986 (0.9955–1.0018)	0.4	1.00054 (0.9933–1.0078)	0.89
Malignant neoplasm of bone and articular cartilage	9	0.33 (0.06–1.83)	0.21	0.25 (0.026–2.45)	0.23	0.08 (0.00047–12.14)	0.35
Mesothelioma	9	0.61 (0.12–3.09)	0.55	0.73 (0.09–5.67)	0.76	2.19 (0.017–279.04)	0.76
Rectum cancer	2	0.9975 (0.9940–1.0011)	0.18	NA	NA	NA	NA
Meningiomas	8	0.93 (0.33–2.58)	0.88	0.65 (0.19–2.21)	0.49	0.71 (0.028–18.015)	0.84
Thyroid cancer	9	1.18 (0.64–2.16)	0.59	1.28 (0.57–2.88)	0.55	1.02 (0.17–6.18)	0.99
Bladder cancer	9	1.00022 (0.9980–1.0025)	0.84	1.00023 (0.9978–1.0026)	0.85	0.9984 (0.9917–1.0052)	0.66

### 3.1. Association of genetic liability to exposures with cancer risks

#### 3.1.1. Univariable MR results

The results of the IVW test revealed a suggestive association of genetic liability to AF and PCA (OR = 0.96; 95% CI 0.92–0.99, *p* = 0.01) (Table 2). However, no relationships of genetic liability to AF with lower odds of breast cancer (OR = 1.003; 95% CI 0.97–1.035, *p* = 0.87), ER-negative breast cancer (OR = 1.009; 95% CI 0.96–1.06, *p* = 0.7), ER-positive breast cancer (OR = 0.99; 95% CI 0.96–1.03, *p* = 0.76), lung cancer (OR = 1.00; 95% CI 0.94–1.06, *p* = 0.97), lung adenocarcinoma (AF: OR = 1.01; 95% CI 0.93–1.10, *p* = 0.76), and squamous cell lung cancer (OR = 1.004; 95% CI 0.92–1.10, *p* = 0.93) were observed. Regarding other 12 site-specific types of cancer, limited evidence validated a causal association of genetic liability to AF with the risk of kidney cancer (OR = 1.0001; 95% CI 0.9996–1.0006, *p* = 0.71), melanoma (OR = 1.0003; 95% CI 0.9999–1.0011, *p* = 0.93), lymphoma (OR = 1.00016; 95% CI 0.995–1.00049, *p* = 0.95), glioma (OR = 1.15; 95% CI 0.97–1.36, *p* = 0.12), colon cancer (OR = 0.9998; 95% CI 0.9992–1.0004, *p* = 0.44), rectum cancer (OR = 1.0002; 95% CI 0.9997–1.00068, *p* = 0.27), meningiomas (OR = 0.95; 95% CI 0.76–1.18, *p* = 0.65), thyroid cancer (OR = 0.95; 95% CI 0.84–1.09, *p* = 0.48), and bladder cancer (OR = 0.9999; 95% CI 0.9996–1.00038, *p* = 0.9); malignant neoplasm of bone and articular cartilage (OR = 0.78; 95% CI 0.54–1.13, *p* = 0.2); and mesothelioma (OR = 1.24; 95% CI 0.85–1.81, *p* = 0.27) (Table 2). Some outliers were observed with the MR-PRESSO analysis, and the results remained in line with the original ones after removing these outliers (Supplementary Table 2). Additionally, we also found no associations between HF and 19 site-specific cancer risks (Table 3).

#### 3.1.2. Multivariable MR analysis

**Figure 3:**
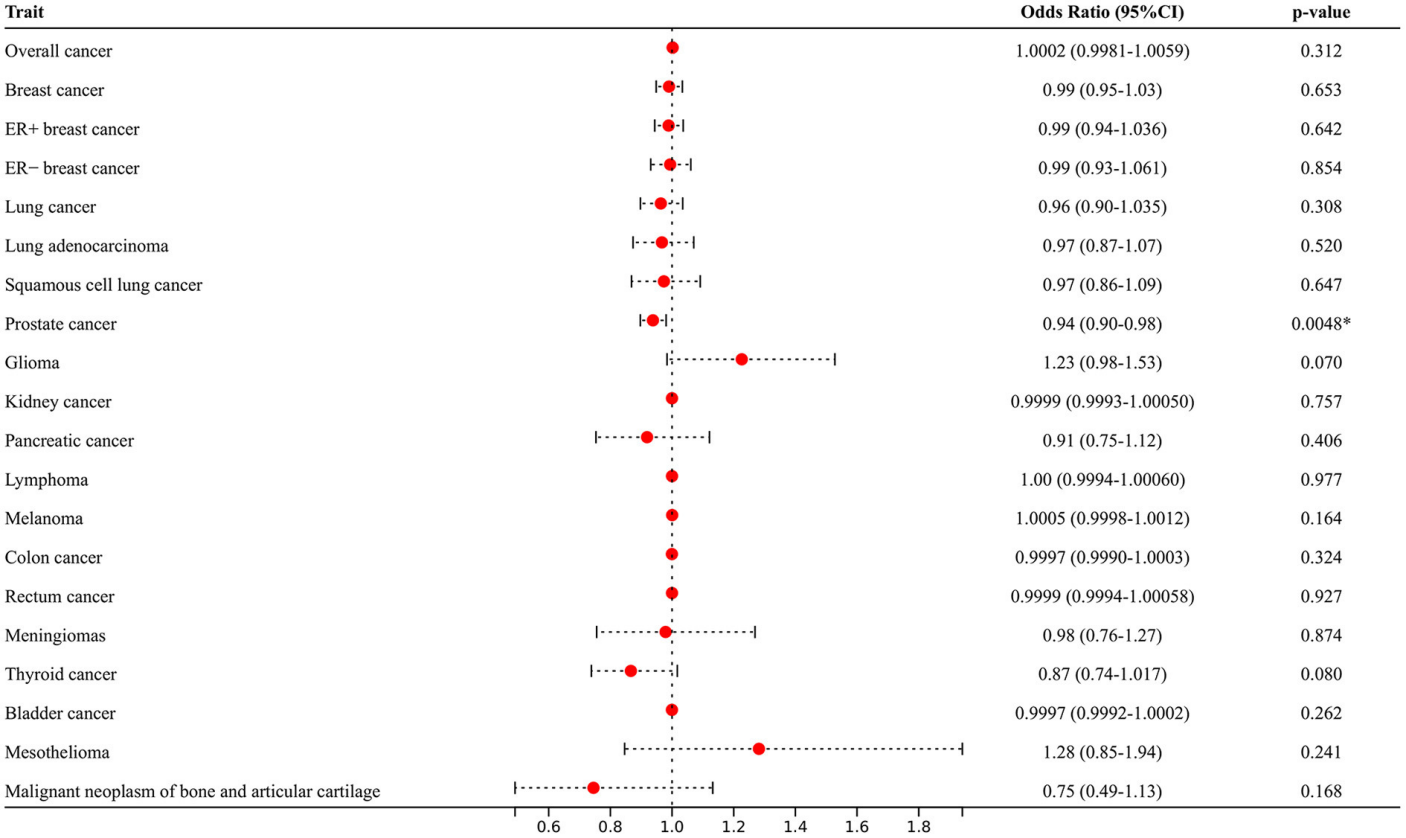
Multivariable Mendelian randomization analyses adjusted for smoking, alcohol consumption, diabetes, and hypertension. ^*^Means statistically significance.

As illustrated in Figure 3 and Supplementary Table 3, after adjusting for potential pleiotropic or mediating effects, multivariable MR still expounded strong independent associations between genetic predisposition to AF and PCA (OR = 0.94; 95% CI 0.90–0.98, *p* = 0.0048) and yielded similar results that AF was not associated with the increased risk of other site-specific cancer types.

### 3.2. Assessment of MR assumptions

The first assumption was met because our included SNPs were selected at the genome-wide significance threshold of p < 5 × 10^−8^ and *F*-statistics values ranged from 273.8 to 369.1 (*F* > 100). Leave-one-out analysis suggested that individual SNPs had no impact on the overall effect of AF on cancer risks. Moreover, the MR-Egger regression analysis suggested that the impact of pleiotropy was negligible because intercepts were not statistically significant (all *p*-values>0.05) (Supplementary Table 4). Sensitive analyses demonstrated that the second MR assumption was not violated. Although the Cochrane *Q-*tests showed certain horizontal pleiotropy, little influence affected the overall results because no pleiotropy biased the results of the MR-Egger and MR-PRESSO tests (32). With regard to the third MR assumption, multivariable MR and MR-PRESSO analyses eliminated pleiotropic effects, which abided by the third MR assumption.

### 3.3. Analysis of the functional characteristics of common DEGs

**Figure 4:**
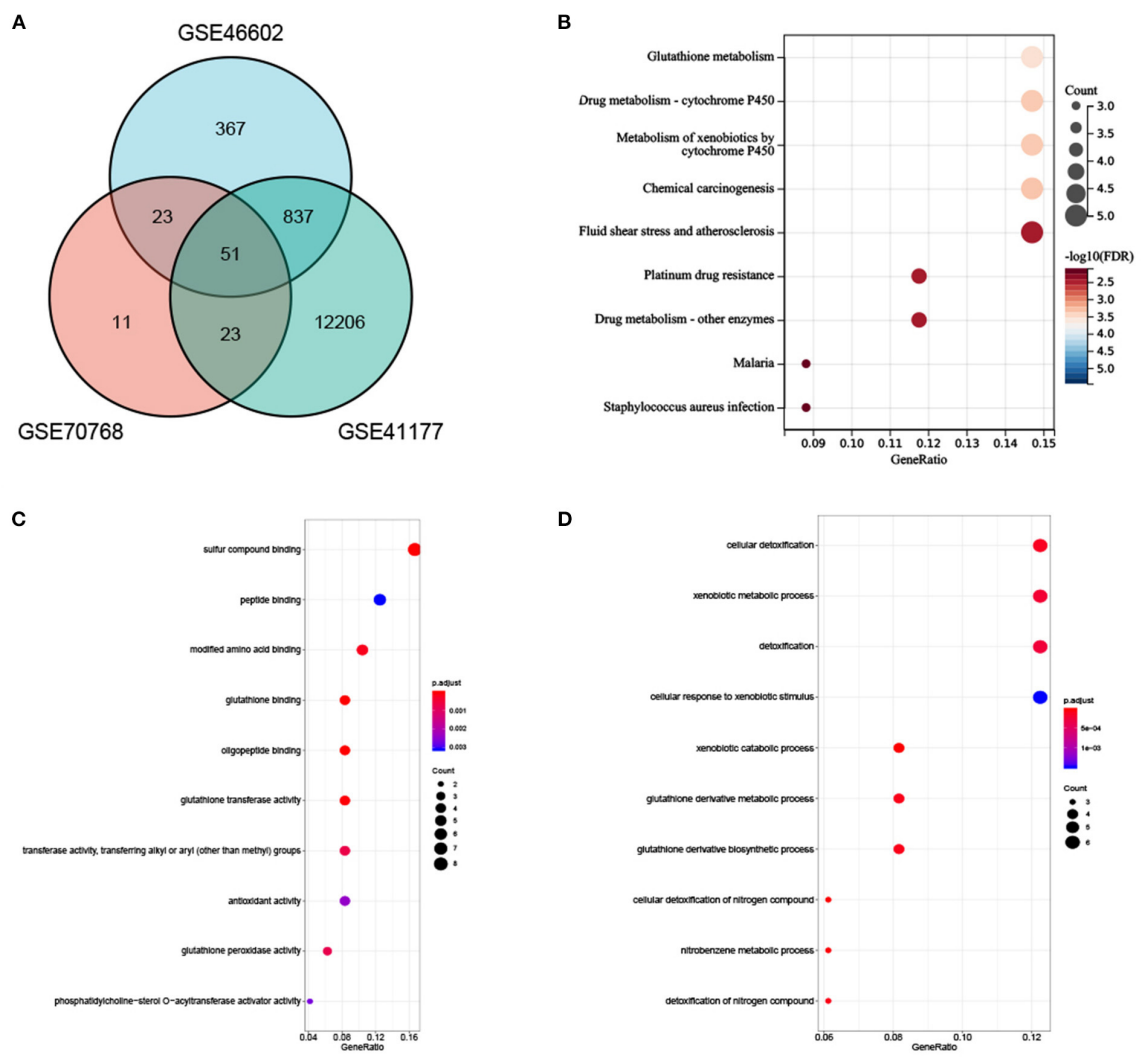
**(A)** Venn diagram showed an overlap of 51 DEGs. **(B)** The enrichment analysis results of KEGG Pathway. **(C, D)** The enrichment analysis results of GO terms.

In total, 51 common DEGs between AF-related and PCA-related datasets were identified (Figure 4A). Results of the KEGG pathway demonstrated that several significant enrichment pathways were noted, such as glutathione metabolism and metabolism of xenobiotics by cytochrome P450 (all *p*-values were <0.05) (Figure 4B). Regarding GO analysis, these DEGs were mainly enriched in cellular detoxification, xenobiotic metabolic process, glutathione derivative metabolic process, cellular response to xenobiotic stimulus, glutathione binding, and antioxidant activity (all *p*-values were <0.05) (Figures 4C, D). These outcomes firmly revealed that the antioxidant activity, xenobiotic catabolic process, and cytochrome P450 metabolism were involved in the development of AF and PCA.

## 4. Discussion

### 4.1. Principal findings

In this study, we performed MR analyses to evaluate whether genetic evidence supported a causal association between AF and the risk of 19 site-specific types of cancer. To the best of our knowledge, this is the first cardio-oncology MR study involving 6,334,916 people to explicate that AF may casually reduce the risk of PCA. Moreover, little evidence demonstrated that AF and closely related HF were casually related to the increased risks of lung cancer, breast cancer, PCA, kidney cancer, glioma, pancreatic cancer, colon cancer, rectum cancer, meningiomas, thyroid cancer, bladder cancer, lymphoma, or melanoma, with less susceptibility to potential confounders and inverse causation. Based on our findings, cancer screening beyond standard routine healthcare may not be currently merited with a new diagnosis of AF. Nonetheless, a tight collaboration between cardiologists and oncologists is still essential to improve the management of patients, which may provide crucial mechanistic and therapeutic insights with regard to both serious conditions.

### 4.2. Previous research

The present MR findings do not support previous observational studies, suggesting that the manifestation of AF was a marker of occult cancer. Several sporadic epidemiological trials have described the underlying effect of AF on site-specific malignancies, but their findings were very controversial. An observational cohort study by Conen et al. (12) included 34,691 women and revealed that women with new-onset AF may have an elevated cancer risk beyond 1 year of AF diagnosis. This questionnaire-derived study also indicated that new-onset AF was statistically significant for the risk of colon cancer, whereas significant multivariable-adjusted relationships for breast cancer were not observed (12). These findings were consistent with a Danish population-based cohort study enrolling 26,222 men and 28,879 women free of AF (8). In this cohort trial, AF was not related to breast cancer or PCA (8), but the risk of colorectal cancer and lung cancer was paradoxically remarkably high within the initial 90 days following the diagnosis of AF (8). A retrospective cohort of 5,130 patients, however, demonstrated that the standard incidence ratio of lung and colon cancer was significantly high in patients with AF, although there was no significant increase in the risks of liver or breast cancer (11). Interestingly, evidence from a prospective cohort study even reached a contrasting conclusion that AF was related to decreased odds of the new diagnosis of breast cancer and colorectal cancer, indicating that an association noted in a previous study may be caused by potential detection bias instead of a causal relationship (9).

Atrial fibrillation and HF are often presented together with each other (16), and a similar scenario also holds for the relationship between HF and cancer risks. Retrospective research has suggested that the prevalence of malignancy in established HF ranges from 18.9 to 33.7 per 1,000 person-years (33–35). Conversely, a study with larger cohorts and longer follow-up durations explicated that HF was neither associated with an increased risk of cancer nor cancer-specific deaths (36). Overall, according to previous studies, an association between AF and malignant tumors has been reported but is largely controversial.

### 4.3. The interpretation of the observed results

The potential limitations in previous epidemiological studies may bias the observed outcomes. The conflicting outcomes regarding cancer risks and AF in prior studies may be mediated by lots of possibilities, and some of the potential relationships are complex.

First, it was not surprising to see elevated cancer risks in patients with AF because related treatments render clinically overt cancer that could be otherwise asymptomatic. Several cardiovascular drugs, including spironolactone (37) and aspirin (38), have been shown to lower the carcinogenesis of certain types of cancer. In addition to the effect of related treatments, inherent drawbacks in observational study design, such as shorter follow-up duration, possible selection/surveillance bias (8, 12), and a lack of comprehensive data on shared risk factors (14, 39), could explain the perplexing results. The median time from AF/HF to malignancy diagnosis in some prior investigations was <3 years (33, 35), which might be too short of a period to expound a causal association. With regard to selection or surveillance bias, some questionnaire-based studies may be unable to accurately determine whether patients underwent the examination before or after AF diagnosis (12). Besides, patients with screen-detected AF are more possible to have cancer screenings at an early stage, which is usually missed in the general population. For instance, if silent malignancies stay undetected, AF-related antithrombotic (40) or anticoagulant agents (8, 12) could increase the positivity rate of intestinal hemorrhage or hematuria, thus followed by several cancer detections (41). Third, AF and cancer are complex conditions and share many common risk factors, including alcohol consumption, smoking, hypertension, and diabetes (42). The risk of malignancy in patients with AF will naturally increase with the presence of the number of these risk factors. Hence, minimizing the effects of confounders and limitations of study designs is necessary for the evaluation of causality.

### 4.4. Possible mechanisms

In the present MR analysis, we found that AF may lower the risk of PCA using univariable and multivariable MR methods. The observed findings may be first attributed to an atrial natriuretic peptide (ANP), which may provide meaningful information about the underlying mechanism. AF is an independent determinant of ANP that exerts an important role in restraining tumor growth (43, 44). The inhibition of malignant cell proliferation by ANP is mediated by both intracellular acidity and Wnt/β-catenin signaling (45). ANP might also hinder the adhesion of malignant cells to microvascular endothelial cells by suppressing the E-selectin expression, which is regulated by inflammation (46). Second, AF-related hypercoagulability would alter cancer cell adhesion and tumor progression by decreasing matrix metalloproteinases (MMPs) in tissue and increasing circulating levels of inhibitors of matrix metalloproteinases (TIMPs) (5). TIMPs could control MMP that could lead to the degradation of the extracellular matrix and, consequently, organize the path for malignant cells to progress and spread to distant secondary areas (47). Third, certain immune-related genes identified in AF have recently been linked to the prognosis and immune infiltration in several tumor types (48). Herein, present KEGG and GO enrichment analyses of 51 common DEGs also revealed that these genes were significantly enriched in antioxidant activity, xenobiotic catabolic processes, and cytochrome P450 metabolism pathways. It has been expounded that the cause of PCA occurrence might be the outcome of an imbalance of antioxidants (49). Antioxidant defenses might be notably attenuated in patients with PCA (50). Moreover, environmental xenobiotics are largely involved in PCA development and are metabolized by cytochrome P450 in the human organism (51).

### 4.5. Strengths and limitations

The present MR study has several notable strengths. First, this is the first MR study conducted to evaluate the causal association between AF and cancer risks. MR analysis is deemed a reliable epidemiological method to evaluate the causality between exposures and outcomes. Residual confounding from unmeasured variations of baseline information may not ascertain cause–effect associations in previous studies (7, 8, 11, 12). The MR analysis, however, may better diminish the interference of confounders and inverse causation. Moreover, we were more likely to portray a relatively independent causal inference from AF to cancer risks with the multivariable MR approach adjusted for confounders. Second, the included AF-associated SNPs as IVs were gained from all documented GWASs, which may better explicate the variation of AF. Third, the present genetic summary data of certain types of cancer were obtained from large-scale consortia (namely, ILCCO, BCAC, and PRACTICAL), including millions of cases, which were far more than some previous studies (7, 11, 12). Compared with the low-occurrence rates of certain tumors in the previous studies (7, 11, 12), the present results from a relatively large sample size and strongly related IVs could present sufficient statistical power and a precise assessment of causal effects.

Some drawbacks should be taken into account to better elucidate the present findings. The participants in our study were of European descent. Thus, the results of our analysis were less likely to be biased by population stratification, but whether our assertion could be generalizable to other populations for different genetic backgrounds needs to be verified. Besides, the sample size of several site-specific cancer types in our analysis was small. For example, the consortium of malignant plasma cell neoplasms consisted of only 180 patients compared with its vast number of 87,061 controls. The statistical power may not estimate their causality accurately. Finally, the association between AF and PCA was not maintained in the results of MR weighted-median and MR-Egger analyses. However, the direction of MR estimates was consistent among IVW, weighted median, and MR–Egger methods in this study. Moreover, MR-PRESSO and multivariable MR tests were conducted to distinguish possible horizontal pleiotropy and supported the original IVW results.

## 5. Conclusion

This large cardio-oncology study revealed that AF may reduce the risk of PCA. Despite the lack of a causal relationship between AF and increased cancer risks, we should not ignore the two diseases' shared risk factors and pathophysiological mechanisms. Numerous studies still investigate the complicated interrelations between AF and cancer stay and, with an aging population, it represents a valuable field for future investigation. A multidisciplinary approach is still needed to better understand the underlying mechanisms regarding the links between AF and cancer risks.

## Data availability statement

The datasets presented in this study can be found in online repositories. The names of the repository/repositories and accession number(s) can be found in the article/Supplementary material.

## Ethics statement

Ethical review and approval was not required for the study on human participants. Written informed consent was not required to participate in this study.

## Author contributions

WL, MH, and WW conceived and designed the study, collected, analyzed, and interpreted the data. The first version of the manuscript was written by WL, RW, and WW. All authors contributed to the article and approved the submitted version.
